# Editorial: Integrative analysis approaches for understanding plant metabolism, metabolite, chemodiversity and their respective regulation

**DOI:** 10.3389/fpls.2023.1248983

**Published:** 2023-07-27

**Authors:** Robin Joshi, Supaart Sirikantaramas, Dinesh Kumar

**Affiliations:** ^1^ Chemical Technology Division, Council of Scientific and Industrial Research (CSIR)-Institute of Himalayan Bioresource Technology, Palampur, India; ^2^ Perelman School of Medicine, Institute for Translational Medicine and Therapeutics (ITMAT), University of Pennsylvania (UPenn), Philadelphia, PA, United States; ^3^ Center of Excellence in Molecular Crop, Department of Biochemistry, Faculty of Science, Chulalongkorn University, Bangkok, Thailand

**Keywords:** metabolomics, chemical diversity, transcriptomics, plant science, metabolism

Plants have specialized metabolic enzymes which evolve rapidly and have multiple functions. Metabolites are important sources of pharmacologically active natural products and refer to specific biological activities associated with their diversified biochemical structures. Metabolites are one of the key parts of defense mechanisms, which are developed by plants in response to biotic and abiotic stresses (Xiao et al., 2022). The identification and quantification of metabolites in different plant organs, tissues, and individual cells, when subjected to various biotic and abiotic stresses provide opportunities for comprehending phytochemistry. These profiles of metabolites can serve as indicators to evaluate the effects of diversified environmental factors on plants and establish protective strategies in agricultural practices (Patel et al., 2021, Wang et al., 2021).

Metabolomics, fueled by advances in high-throughput mass spectrometry, has emerged as a key tool in systems biology, enabling comprehensive and sensitive analysis of the metabolome, encompassing hundreds to thousands of metabolites and their chemo-diversity. Additionally, the rapid progress in high-throughput sequencing has facilitated the utilization of omics technologies, such as genomics, transcriptomics, and proteomics, to gain additional perspectives to understand the mechanisms of interaction within a specific system. Furthermore, when integrated with metabolomic studies, omics-based investigations can progressively elucidate the metabolite accumulation pathways and stress alleviation in plants. Therefore, invitations were sent to outstanding experts in this field to contribute their manuscripts to this Research Topic. An attempt was made to understand to explore metabolites, their diversity, and respective regulation using an integrated omics approach. Finally, a total of 43 authors contributed and 5 research articles were published in this Research Topic.

Pu et al. contributed on elicitor-induced camptothecin (CPT) biosynthesis in *Camptotheca acuminata* plantlets using an integrated approach of untargeted metabolomics and transcriptomics. MeJa, AgNO_3_, and PEG were the main elicitors used to produce CPT biosynthesis, which increased by up to 78.6, 73.3, and 50.0%, respectively. Mass spectrometry helped to identify 15 new alkaloids and 25 known CPT analogs and other downstream precursors. This study explored the CYP450-mediated oxidation steps for CPT biosynthesis and their regulation using an omics approach. Camptothecins are natural alkaloids having various biological effects such as anti-viral activity, anticancer, antiparasitic, and antitumor activity (Liu et al., 2015). Camptothecins will surely benefit from this study on their bioactivities in the near future. 

In another study, transcriptome and metabolome disclosed the key genes involved in phenylpropanoid and flavonoid biosynthesis in an important aquatic cash crop *Trapa bispinosa* by (Yin et al.) Matured samples of shell, leaf, root, and stem were harvested and compared using transcriptomics and metabolomics. The shell and leaf group exhibited the greatest quantity of specific genes participating in the phenylpropanoid, flavonoid, flavone, and flavonol biosynthesis pathways as compared to root and stem. These results open new avenues for investigation into the molecular processes and functional characterization of *Trapa bispinosa* candidate genes for phenolics production. These findings point to the need for further research on phenolic biosynthesis in *Trapa bispinosa*.

A significant variation in pericarp thickness in *Camellia drupifera* and *Camellia oleifera* was investigated through transcriptome and metabolome analyses (Li et al.). Camellia fruit is a woody edible oil source with a recalcitrant pericarp. Fruit samples of both trees were collected randomly from different branches and stored at -80°C followed by RNA and metabolites extraction. Metabolomics examination determined 40 differentially expressed metabolites through UHPLC-Q-TOF-MS. A total of 29 genes were differentially contributed to lignin biosynthesis and evaluated using KEGG and co-expression analysis. Transcription factors NAC and MYB were significantly involved in major transcriptional regulatory mechanisms. High lignin content is the main factor for fruit pericarp thickening. S-lignin accumulated in the pericarp assisted by nine upregulated genes. These results explained the molecular features of pericarp thickening. This study also gives insight into the growth-defense mechanism and seed accessibility in *C. drupifera*. The defense mechanism will be helpful to determine the phenolic biosynthesis in *Camellia drupifera* and *Camellia oleifera*.

Zhao et al. revealed tobacco leaf metabolites using untargeted metabolomics and sensory evaluation studies. These studies evaluated the comparative approach to determine the sensory traits of tobacco products by an expert panel at 350°C with combustible tobacco. Thirteen volatile and 345 non-volatile compounds were identified using non-targeted metabolomics studies. The sensory quality of heated tobacco predicted that *β*-damascenone, scopoletin, chlorogenic acids, neochlorogenic acids, and flavonol glycosyl derivatives had strong contributions. Lyso-phosphatidylcholine, lyso-phosphatidylethanolamine, and reducing and non-reducing sugar molecules were also linked positively to sensory quality. The authors described the positive relationship between volatile and non-volatile metabolites. These discriminations provide insights into the chemical predictors for selecting tobacco varieties with desired sensory quality. By understanding the chemical composition and sensory impact of different tobacco types, researchers can make informed decisions regarding the selection and breeding of tobacco varieties to meet desired sensory preferences.

In the last manuscript, sugar metabolism plays an important role in plant growth and development as well as stress tolerance (Cheng et al.). Sucrose degradation is mainly catalyzed by an invertase enzyme. A deep genomics study revealed 20 alkaline/neutral INV genes (NtNINV1-20), 4 vacuolar INV genes (NtVINV1-4), and 12 cell wall INV isoforms (NtCWINV1-12) in *Nicotiana tabacum*. Decreased level of fructose and sucrose was observed in *NtNINV10* silenced tobacco gene, which has a potential role in sugar metabolism, growth, and development in plants. This study is helpful in understanding the role of INV genes in leaf maturation and development. The present study has broader implications for plant breeding and crop improvement strategies aimed at enhancing plant productivity and overall performance.

In conclusion, the present Research Topic significantly advances the frame of knowledge on an integrated approach to understanding plant metabolism, metabolites, chemo-diversity, and their respective regulation under stresses. As an editor, I would like to thank all the contributing authors, reviewers, and editors of Frontiers in Plant Sciences for their assistance.

## Author contributions

All the mentioned authors contributed significantly and intellectually to the work and granted their consent for it to be published.
